# Pharmacy network and access to medicines in selected eastern European countries: comparative analysis

**DOI:** 10.3325/cmj.2012.53.53

**Published:** 2012-02

**Authors:** Dragana Lakić, Ljiljana Tasić, Mitja Kos, Guenka Petrova, Assena Stoimenova, Dušanka Krajnović

**Affiliations:** 1 University of Belgrade, Faculty of Pharmacy, Department of Social Pharmacy and Pharmacy Legislation, Belgrade, Serbia; 2 Chair for Social Pharmacy, Faculty of Pharmacy, University of Ljubljana, Ljubljana, Slovenia; 3 Department of Social Pharmacy, Faculty of Pharmacy, Medical University of Sofia, Sofia, Bulgaria

## Abstract

**Aim:**

To analyze the pharmacy network (structure and resources) in Bulgaria, Croatia, Serbia, and Slovenia and its relation to public expenditures for medicines.

**Methods:**

We performed a cross-sectional study using the officially published data for the period 2003-2008 in four selected countries. Data sources were relevant national institutions.

**Results:**

In 2008, Serbia had 27.5, Bulgaria 66.8, Croatia 59.5, and Slovenia 71.2 pharmacists per 100 000 inhabitants. There was a significant difference in the number of pharmacists per 100 000 inhabitants between all countries except between Bulgaria and Slovenia. The number of inhabitants per one pharmacy was significantly different between all observed countries. The expenditures for medicines per capita in 2008 were between €30.34 in Bulgaria to €137.03 in Slovenia, with a significant difference between all countries except between Bulgaria and Serbia. The number of pharmacists per 100 000 inhabitants and expenditures for medicines per capita were positively correlated in all observed countries, except in Bulgaria.

**Conclusion:**

There were significant difference in the structure and availability of the pharmacy service in all selected countries. Expenditures for medicines were positively correlated with the number of pharmacists in all countries, except in Bulgaria. Our findings could be valuable to national regulatory bodies for the creation of national drug policies.

Regular access to medicines is still a problem for some countries in Europe (1). Access to medicines is a complex concept, consisting of the dimensions of availability, affordability, and accessibility. Availability, defined as type and quantity of health technology needed or provided, highly relies on the availability of health care professionals and health infrastructure. Affordability is defined as the cost to the patient or society imposed by health technology and accessibility as access to quality health care, in terms of the adequate number of health professionals and health facilities (2). In case of the pharmaceutical sector, all of this refers to adequate resources and resource allocation. Resources include humans, facilities, and financial resources. In plain words, it refers to geographical network of pharmacies and pharmacists, as well as optimal medicine financing (2,3). There is a limited number of studies investigating pharmacy network and access to medicines in Central Eastern European (CEE) countries. The study from 2003 reveals a shortage of pharmacists, which could have a negative impact on pharmacy services and their quality (4). The information about the number of pharmacies is inconsistent. Data on the number and ownership type of pharmacies are published for few countries, with very scant information for the CEE region (4-7).

Development of pharmacy network and access to medicines highly depends on the overall functioning of the health care system. Central issue in any health care system is the type of financing. CEE countries use mandatory social health insurance (often called Bismarckian). Funds are collected from the insured persons as percentage of their salary. This type of health insurance allows the coverage of almost 100% of the population. Further revenue comes in the form of cost sharing for the services covered by the benefits package. Cost sharing commonly applies to outpatient prescription medicines and depends on their life-saving potential, relative therapeutic value, and price.

The aim of the study was to analyze the pharmacy network in terms of the number of pharmacies and pharmacists in four CEE countries – Bulgaria, Croatia, Serbia, and Slovenia and to measure the access to medicines through the public expenditures for medicines, and correlation between these indicators. We tried to evaluate if citizens of the selected countries had equal access to pharmacy services and reimbursed medicines, and compare the results with other European countries.

## Materials and methods

### Design and methodology

We conducted a cross-sectional study in Bulgaria, Croatia, Serbia, and Slovenia. These countries were selected because their pharmaceutical sector was a centralized market until 1990 after which changes in its structure and financing occurred. All the selected countries have just one public health insurance fund, similar socio-economic development, and similar public health expenditures (as percent of gross domestic product).

Pharmacy network and access to medicines was evaluated using the following indicators: number and type of pharmacies, number of inhabitants per one pharmacy, number of pharmacists, public expenditures for medicines, and public expenditures for medicines per capita. The number and distribution of pharmacists and pharmacies were factors important for availability, while organization (types of facilities) and financing (cost sharing and co-payment arrangements) were important for accessibility, affordability, and quality of health care system (3,8).

### Data sources

We used the officially published data for the period 2003-2008. Population data for all 4 countries were obtained from the database of national institutes for statistics (9-12). All community pharmacies were included in the analysis and hospital pharmacies were excluded since they are not directly available to out-patients (3). In Bulgaria, the number of publicly owned and private pharmacies, number of pharmacists, and public expenditures for medicines were obtained from the Ministry of Health database (13). In Croatia, the number of pharmacists and the number and type of pharmacies were obtained from the Croatian National Institute of Public Health (14). In Serbia, the number of pharmacists and number of private pharmacies were obtained from the Statistical Office of the Republic of Serbia (11) and the number of public pharmacies was obtained by a telephone interview with each of the 29 regional pharmacy institutions. In Slovenia, the number and type of pharmacies was obtained from the database of the Slovenian Chamber of Pharmacy (15) and the number of pharmacists was obtained from Slovenian Institute for Public Health (16).

Expenditures for medicines data were obtained from the Croatian Institute for Health Insurance (17), Serbian Institute for Health Insurance (18), the Health Insurance Institute of Slovenia (19), and the Ministry of Health of Bulgaria regarding the payments for life saving medicines and from the National Health Insurance Fund for all other reimbursed medicines. All expenditures are presented in Euro, using the following average exchange rates: Bulgaria €1 = BGN 1.95; Croatia €1 = HRK 7.39, and Serbia €1 = RSD 79.79).

### Statistical analysis

Mean values of the number of pharmacists per 100 000 inhabitants and the number of inhabitants per one community pharmacy were compared between the four countries with one-way ANOVA. Testing for significant differences between groups was performed with Tukey HSD post-hoc test. The effects of the number of pharmacists and number of pharmacies per 100 000 inhabitants on public expenditures per capita were tested by linear regression analysis. A two-tailed *P* ≤ 0.05 was considered significant. All calculations were performed using STATGRAPHIC Plus, version 4.2 software (Statpoint Technologies Inc., Warrenton, VA, USA).

## Results

**Figure 1:**
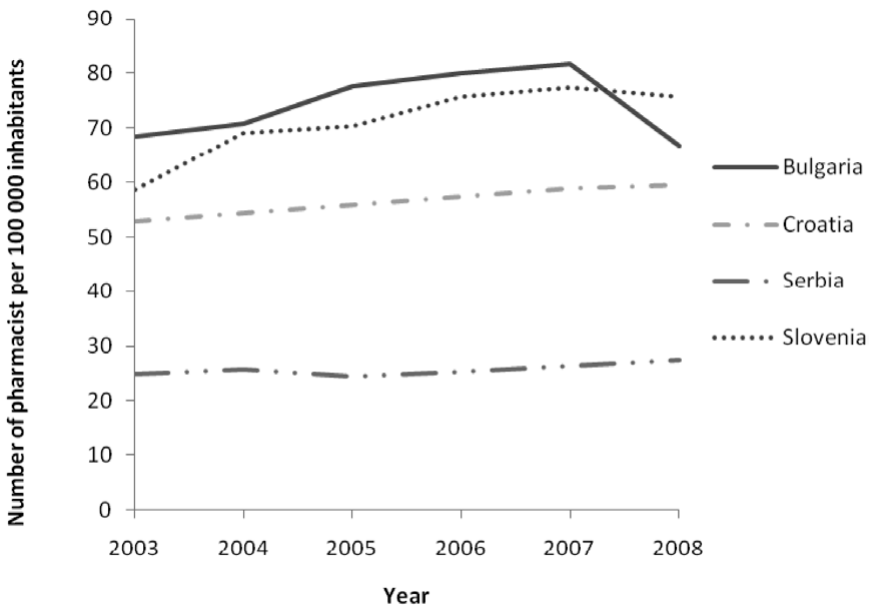
Number of pharmacists per 100 000 inhabitants in Bulgaria, Croatia, Serbia, and Slovenia in the period 2003-2008.

**Figure 2:**
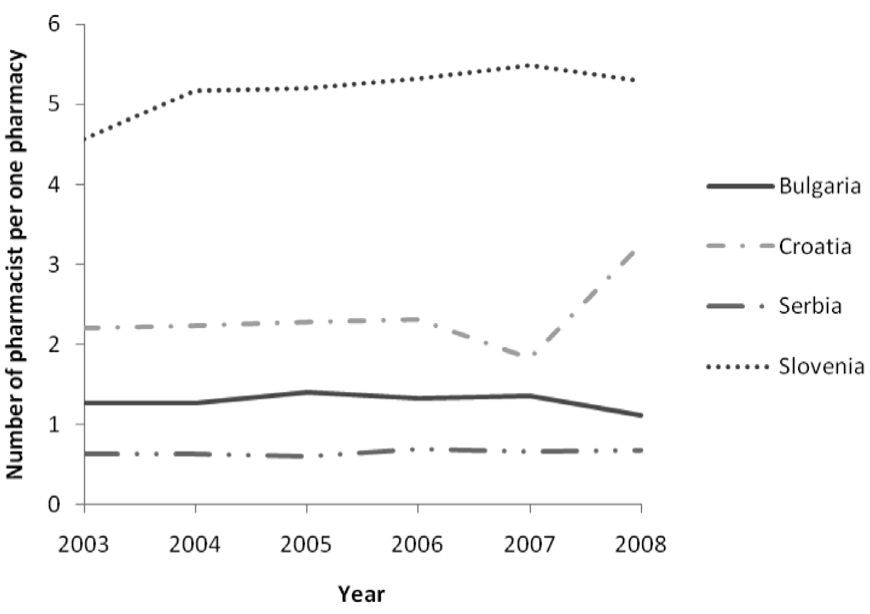
Number of pharmacists per one pharmacy in Bulgaria, Croatia, Serbia, and Slovenia in the period 2003-2008.

All countries had two or more times more pharmacists per 100 000 inhabitants than Serbia. In 2008, Serbia had only 27.5 pharmacists per 100 000 inhabitants, while Bulgaria, Croatia, and Slovenia had 66.8, 59.5, and 71.2 pharmacists, respectively (Figure 1). A significant difference in the number of pharmacists per 100 000 inhabitants was observed between all countries (F = 122.89; *P* < 0.001 ANOVA with Tukey post-hoc test), except between Bulgaria and Slovenia. The greatest number of pharmacists per pharmacy was observed in Slovenia (5.29 pharmacists per pharmacy in 2008), while Serbia in 2008 had just 0.67 pharmacist per pharmacy, although one pharmacist per pharmacy is the minimum legal requirement (Figure 2).

**Figure 3:**
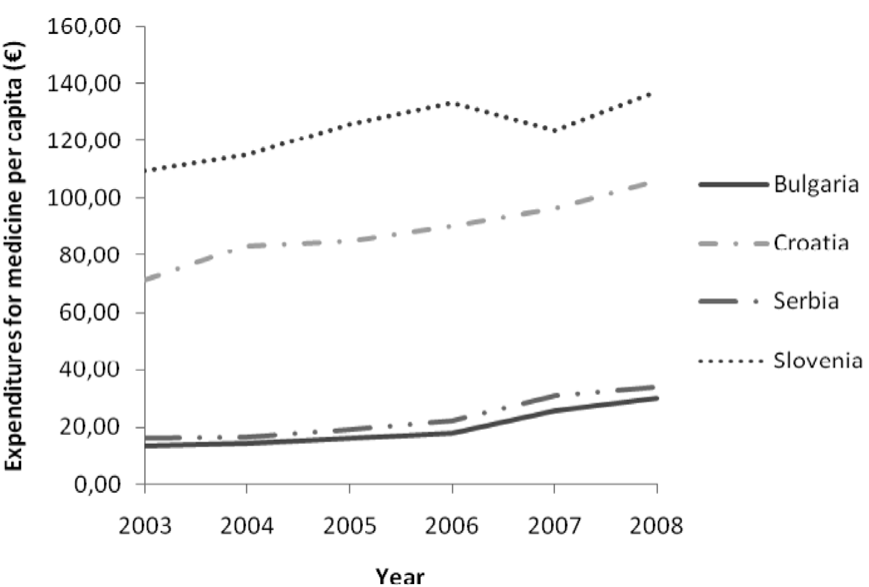
Public expenditures for medicine per capita for Bulgaria, Croatia, Serbia, and Slovenia in the period 2003-2008.

In Bulgaria, all community pharmacies (n = 4557 in 2008) were privately owned (either independent or part of a pharmacy chain). In Croatia, the number of privately owned pharmacies gradually grew from 321 in 2003 to 445 in 2008, reaching a total of 55% of all pharmacies. In Serbia, the majority of pharmacies (around 80% of 2995 in 2008) were privately owned, but only publicly owned pharmacies (590 in 2008) were allowed to dispense reimbursed medicines according to the contract with Republic Institute for Health Insurance. In Slovenia, the number of pharmacies remained almost constant during the observed period and the majority of them (approximately 70%) were publicly owned. The greatest number of inhabitants per pharmacy was in Slovenia, with approximately 7000 inhabitants (Table 1). The number of inhabitants per one pharmacy was significantly different between all countries (F = 210.66; *P* < 0.001). Public expenditures for medicines per capita in 2008-year ranged between €30.34 in Bulgaria to €137.03 in Slovenia (Table 2). There was a significant difference in public expenditures for medicines between all countries except between Bulgaria and Serbia (F = 233.64; *P* < 0.001) (Figure 3).

**Table 1:** Number of inhabitants per pharmacy in Bulgaria, Croatia, Serbia, and Slovenia (year 2003-2008)

	2003	2004	2005	2006	2007	2008
Bulgaria	1858	1793	1816	1658	1659	1669
Croatia	4185	4118	4083	4022	3102	5459
Serbia	2584	2442	2444	2753	2501	2454
Slovenia	7769	7479	7384	7023	7086	6984

**Table 2:** Public expenditures for medicine per capita in Bulgaria, Croatia, Serbia, and Slovenia in year 2008

Country	Public expenditures per capita (€)
Bulgaria	30.34*
Croatia	105.80*
Serbia	34.16*
Slovenia	137.03

*Conversion rates: Bulgaria €1 = BGN 1.95; Croatia €1 = HRK 7.39; and Serbia €1 = RSD 79.79.

Correlation analysis between the number of pharmacist per 100 000 inhabitants and public expenditures for medicines per capita showed a positive correlation for all observed countries, except Bulgaria. The number of pharmacists explained more than 93% of the variation in expenditures in Croatia (b = 4.47, r = 0.968, *P* = 0.001); 71% in Serbia (b = 5.82, r = 0.847, *P* = 0.033), and 70% in Slovenia (b = 1.24, r = 0.818, *P* = 0.046). When we compared pharmacies instead of pharmacists, univariate regression analysis showed a significant correlation between the number of pharmacies and expenditures for medicines only in Slovenia (b = 16.67, r = 0.914, *P* = 0.011).

## Discussion

Our study showed that all investigated countries had different number of responsible pharmacists and that Serbia had significantly fewer pharmacists than Bulgaria, Croatia, or Slovenia. Serbia had almost three times fewer pharmacists than Bulgaria or Slovenia and two times fewer than Croatia. We used the number of inhabitants per one pharmacy as the indicator of accessibility to medicines and other pharmacy services (2). The smallest number of inhabitants per pharmacy was observed in Bulgaria, while Slovenia had more than 4 times higher value. Due to high number of inhabitants per pharmacy, Slovenia seems to have the lowest accessibility to pharmacy services, but on the other hand it has the highest number of pharmacists per pharmacy. Also, Croatia had a large difference in the number of inhabitants per pharmacy between 2007 and 2008, which could be explained by huge decline in the number of publicly owned pharmacies in 2008 (almost by 50%). The study also found big differences in public expenditures for medicines between the countries and a positive correlation between these expenditures and the number of pharmacists.

In Europe (27 EU member states, Croatia, FYR Macedonia, Norway, Switzerland, and Turkey) in 2006, the average number of responsible pharmacists per community pharmacy was 2.4. At the same time, there were 176 053 community pharmacies, which corresponds to an average of 3300 inhabitants per pharmacy (20). A possible reason for the small number of pharmacists in Serbia could be a low socioeconomic status and migration of health workers. Migration of health workers from the new to old EU members has been previously noticed (21,22), and further migration could be expected from Croatia and Serbia after their accession to the EU. In Europe, the average number of pharmacists per 100 000 in 2006 was 52.28, while in the EU it was 71.43 (23), ranging from 17 per 100 000 in the Netherlands to 89 per 100 000 in Ireland (8). The smallest number of pharmacists per 100 000 in the world (only 1) is observed in some countries of Africa and the Middle-East (24,25).

Pharmacists play an important role in the prevention and treatment of diseases. In the 29-professions survey in Australia, they were placed among the most ethical and honest professions (26). Often, they represent the only point of contact with the health care system. Even with the adequate number of pharmacists, the average time for advice and counseling in pharmacy is about 2 minutes (27), which means that in countries with a low number of pharmacists (like Serbia) the quality of pharmacy services, especially pharmaceutical care, is questionable. Serbia had just one pharmacist per 4000 inhabitants during the observed period, while Slovenia had one pharmacist per 1300 inhabitants.

There were great differences in the number of inhabitants per community pharmacy between countries. The reasons for these differences may be regulatory – pharmacies in Slovenia is are owned exclusively by licensed pharmacists, which is not the case in Bulgaria (28,29). They may also be geographical – in Slovenia the distance between two pharmacies must be at least 400 m, with the number of inhabitants covered by a pharmacy ranging from 5000 to 7000 (30), while the Bulgarian pharmacy network is overdeveloped so there are often many pharmacies in an area of only a few hundred square meters (31). This could also be related to pharmacy infrastructure (possibility the existence of a few, large pharmacies in Slovenia). Denmark has the greatest number of inhabitants per pharmacy in the EU (6) and Greece has the smallest (32). EU average in 2006 was about 3300 inhabitants per pharmacy, with 2.4 responsible pharmacists per pharmacy (20).

The number of pharmacies is associated with multiple market factors (33). In Serbia, reimbursable medicines are dispensed only by publicly owned pharmacies, since only they have contracts with the Serbian Health Insurance Institute. For years, private pharmacies are trying to become integral part of this network, but with little effect. If the number of inhabitants per pharmacy is calculated solely on the basis of publicly owned pharmacies, it would be almost 13 000. Since the number of pharmacists per pharmacy in Serbia is small, accessibility of pharmacy services and medicines could be jeopardized. A possible explanation for the small number may lay in the fact that although pharmacists’ licensing became obligatory in 2008 (34), the inspections by Ministry of Health were rare due to a small number of inspectors.

The differences in public expenditures for medicines per capita in the observed countries may be influenced by differences in legislation, mainly size of contribution to the national insurance funds (15% in Croatia, 13.45% Slovenia, 12.3% in Serbia, and 6% in Bulgaria) (35-37); pharmaceutical regulatory aspect (cost containment measures for pricing and reimbursement of medicines, different wholesale and retail margins, and difference in value added tax for medicines), and out-of-pocket spending (in Bulgaria 44.8% of total health care expenditures was allocated on out-of-pocket spending in 2004 and in Slovenia only 9.7%) (36). Regulatory policies in CEE countries are trying to reduce costs through restrictions in prescribing high volume or expensive medicines (38-40).

Expenditures for medicines are one of the fastest growing components of the total health expenditures and are ranked third after the hospital and ambulatory health care expenditures (41). The increase is attributed to greater demand, aging population, and innovative, expensive medicines. There is a lack of data on average per capita expenditures for medicines in Europe (7); Organization for Economic Co-operation and Development (OECD) countries had average expenditures of USD 446 in 2006, meaning that all analyzed CEE countries were far below the OECD average. A previous study confirmed that other CEE, post-2004 EU member countries had lower expenditures for medicines per capita, eg, Poland GBP 66, than pre-2004 EU countries (42).

Interestingly, only Bulgaria had a weak negative association between the number of pharmacists and public expenditures per capita. In Bulgaria, the number of pharmacists declined in 2008 after all pharmacists practicing their profession had been required to register (43). Hence, pharmacists in pharmaceutical companies, representative offices, and similar did not register, which could explain the weak negative correlation and the small correlation coefficient.

Our study dealt only with the pharmacy sector, which led to certain limitations. First, it did not include physicians, although several studies found that physicians were one of key elements that affected the utilization of reimbursed medicines (44,45). Second, we used the database on the utilization of prescription medicines rather than on all authorized medicines dispensed through pharmacies, and this might affect the overall results of the study. In Serbia, there is a considerable difference in the data on utilization of medicines obtained from the Institute for Health Insurance, which gathers data on prescription medicines, and those obtained from the Medicines and Medical Devices Agency, which gathers all medicine consumption data. The Agency gathers data on annual consumption of their authorized medicines by pharmaceutical companies or representative offices, which reflects only the volume of produced or imported medicines rather than that of medicines actually used by patients (46-49). Also, utilization databases of all market-approved medicines are not available in every studied country. Finally, we did not explore the availability of particular key medicines (for example, statins, proton pump inhibitors, or biotechnology medicines).

In conclusion, there are great differences in the development of pharmacy sector in the selected countries. The availability of pharmacy service seems to be sufficient in Croatia and Slovenia, but could be insufficient in Serbia due to small number of pharmacists. Access to medicines is increasing in all CEE countries; however, it is still lower than in more economically developed EU countries. Our findings could be valuable to national regulatory bodies and could be used in the creation of national drug policies. It would be beneficial to further evaluate the availability of selected medicines (eg, brand medicines and generics).
